# Small-Pox or Tuberculosis

**Published:** 1921-03-12

**Authors:** 


### Small-Pox or Tuberculosis.

It will be remembered that long ago, when sanatorium treatment of consumption came in, recourse was had for institutional accommodation in some instances to small-pox hospitals that, happily, had come to stand constantly empty. Some misgivings were entertained at first as to their possible speedy clearance in face of being suddenly wanted for their original purpose. However, this practically never happened, and nearly all anxiety became allayed. Moreover, the war came, with a great increase in tubercle, but none in small-pox, so that the conversion of accommodation for the latter disease into that for the former, especially as labour, material, and money for new building were lacking, was urgently required.

### First Signs of Danger.

Economy in hospital accommodation space had never been half so necessary before, and small wonder that nothing was done to provide for an emergency that had seemed so little imminent in the past, and likely to be even less so for the immediate future. For, of course, compulsory adult vaccination of great masses of the population for the first time promised security against any immediate danger of variola. However, the war period has now passed, and the danger feared to begin with has shown itself in at least two places in the country. Only to a very slight extent, it is true, and in dimensions quite manageable and unformidable, but still so as obviously to demonstrate that one cannot have the cake and eat it too, cannot grow two trees on the same square yard, and cannot provide for small-pox and tuberculosis both with the same building. In one of these instances a sixty-bed sanatorium for consumptives has recently been cleared of its inmates (who were given a week's notice) pending the development of contact cases with some dozen small-pox patients from the town which it serves. The other instance is at Glasgow, where the Robroyston Hospital, originally intended for small-pox, and since fitted out with over 400 beds for phthisis, and with a staff of eighty officials, is now devoted to the treatment of about a dozen small-pox patients, at a cost, as the local newspapers characteristically mention at the very outset of their remarks, of about £5 per patient per day. This state of things is made more serious by the information we gather next, that at December 31 last there were 182 tuberculous persons on the waiting-list—111 for admission to hospitals, and 71 to sanatoria. During 1920, too, deaths from phthisis numbered 1,155; from small-pox but 110. There appears to be another hospital at which small-pox was formerly treated, but which is now used as an ordinary fever hospital. This is not in a very isolated position, and of course variola is a formidable disease to keep separate from scarlatina, measles, etc. Still, under the pressing circumstances outlined above there is small cause for wonder at the action taken by the Health Committee, which is to transfer the twelve small-pox patients, who so scantily populate Robroyston, over to the fever hospital, by name Belvidere—strange cognomen for a Glasgow edifice!

### A Possible Solution.

Surely to prevent such excursions and alarums is a very simple thing! Buy or rent a field; buy two Army huts; fit heating, lighting, water, and drainage apparatus; and tell your local fever hospital authorities to be prepared to mobilise a flying column to staff it, bringing down the equipment with them, at a day's notice. But, though simple, this plan has one great drawback. It would involve giving your medical superintendent and your matron some authority and initiative. And at that why all the officiousness of the *rentier* (and *rentière*) who are found so much too often still on municipal committees would be up in arms! This concluding remark is, of course, purely general, and without the slightest reference to any particular locality, least of all to our friends in the second city in the kingdom, whose method of dealing with the difficulty we certainly approve.

